# Agreement Between the OptoGait and Instrumented Treadmill System for the Quantification of Spatiotemporal Treadmill Running Parameters

**DOI:** 10.3389/fspor.2020.571385

**Published:** 2020-10-23

**Authors:** Amy N. Weart, Erin M. Miller, Gregory M. Freisinger, Michael R. Johnson, Donald L. Goss

**Affiliations:** ^1^Department of Physical Therapy, Keller Army Community Hospital, West Point, NY, United States; ^2^Baylor University – Keller Army Community Hospital Division 1 Sports Physical Therapy Fellowship, West Point, NY, United States; ^3^Department of Civil and Mechanical Engineering, United States Military Academy, West Point, NY, United States; ^4^Department of Physical Therapy, High Point University, High Point, NC, United States

**Keywords:** gait analysis, running, spatiotemporal parameters, OptoGait, method comparision

## Abstract

The measurement of spatiotemporal gait parameters is commonly utilized to assess gait in healthy and injured individuals. The OptoGait system is a portable system and can be mounted to a treadmill to collect data in a clinical, training, or research setting. The purpose of this method comparison study was to examine the agreement of spatiotemporal gait parameters calculated by the OptoGait compared to an instrumented treadmill system during running. Thirty healthy runners ran on an instrumented treadmill with the OptoGait 1-m system mounted along the treadmill platform. Spatiotemporal running variables of step rate, step length, and contact time were calculated during the final minute of treadmill running. The level of agreement between the OptoGait and treadmill was analyzed using intraclass correlation coefficients [ICC (2,3)] for step rate, step length, and contact time. Step rate and step length demonstrated excellent agreement. Contact time demonstrated good agreement. Intraclass correlation coefficients for spatiotemporal parameters ranged from 0.83 to 0.99. The OptoGait demonstrated good to excellent agreement in the evaluation of running step rate, step length, and contact time and should be considered for use in clinical, training, or research settings.

## Introduction

The measurement of spatiotemporal gait parameters is commonly utilized to assess gait in healthy (Hollman et al., 2011) and injured (Maffiuletti et al., 2009) individuals. More recently, running retraining has received increased attention in the literature and clinical practice as a focused intervention to reduce the biomechanical risk for injury. Spatiotemporal parameters during running have been linked to biomechanical risk factors for running related injuries (Bredeweg et al., 2013; Schubert et al., 2014). Previous studies have successfully altered lower extremity biomechanics during running by altering step rate, step length, or contact time (Edwards et al., 2009; Heiderscheit et al., 2011; Wellenkotter et al., 2014; Adams et al., 2018). Therefore, it is clinically important for clinicians to be able to feasibly and validly measure running spatiotemporal parameters to inform treatment. Instrumented treadmills are considered a valid measurement device for assessing certain running parameters (Kluitenberg et al., 2012). While valid, these treadmills are costly, require large custom spaces for operation, trained personnel to operate, and in some instances require custom code to calculate variables of interest, making this method not ideal for clinical and training settings.

The OptoGait is a portable system and can be mounted to a treadmill to collect data. OptoGait uses high-density photoelectric cells between transmitting and receiving bars, which detect interruption in light signals to automatically calculate spatiotemporal parameters. The automatic calculation of gait parameters is clinically desirable and efficient.

OptoGait has demonstrated excellent reliability for variables of step rate, step length, and contact time during treadmill (Lee M. et al., 2014) and over ground walking (Lienhard et al., 2013; Lee M. M. et al., 2014) as well as good test-retest reliability (Lee M. M. et al., 2014; Gomez Bernal et al., 2016) in healthy and injured adults. To our knowledge, the level of agreement of the OptoGait system during treadmill running compared to an instrumented treadmill has not been published. Running mechanics differ from walking to include increased step rate, increased step length, and decreased contact time (Nuesch et al., 2018). The purpose of this method comparison study was to examine the level of agreement of spatiotemporal gait parameters calculated by the OptoGait system compared to an instrumented treadmill system during running.

## Methods

### Participants

Thirty healthy recreational runners (mean age 30.8 ± 9.8 years; 24 males, 6 females; height 1.8 ± 0.1 meters; mass 80.0 ± 11.6 kg; mean weekly run distance 17.7 ± 17.4 km) volunteered to participate in the study. All participants met the following criteria: (1) Department of Defense Beneficiary (Active Duty Soldier, Cadet, or military dependent) between the ages of 18–60 years; (2) Run at least a 2.7 meters per second (m/s) pace for 5 min; (3) No recent history of lower extremity or back injury within the previous 3 months or surgery within the previous 6 months; (4) Not currently under a running restriction by a medical provider; (5) Not currently pregnant or pregnant within the previous 4 months; (6) Able to read and speak English to give consent and follow study instructions. To eliminate double limb support indicative of walking gait, a minimum pace of 2.7 m/s was used to define running gait. This pace is consistent with previous treadmill running protocols (Asmussen et al., 2019). The study protocol was approved by the Keller Army Community Hospital Institutional Review Board, and written consent was obtained prior to participation.

### Procedures

All participants ran for 5 min at a self-selected pace (mean 3.2 ± 0.3 m/s) on an instrumented treadmill (Bertec, Columbus, OH, USA) with the OptoGait 1-meter system (OptoGait, Microgate, Bolzano, Italy) mounted along the treadmill platform. Self-selected pace was defined as a pace similar to a 2-mile run for exercise (Queen et al., 2006). The Bertec instrumented treadmill demonstrates comparable force traces to over ground running (Asmussen et al., 2019) and thus was chosen as the gold standard. The OptoGait consisted of a transmitting and receiving bar with each bar containing 96 LED diodes that are positioned 1 centimeter (cm) apart and 3 millimeters (mm) above the base. The OptoGait was calibrated according to manufacturer's guidance. The treadmill running test was selected from the predefined OptoGait tests. The OptoGait user input the treadmill speed into the OptoGait system based on the participant's self-selected pace.

Raw force data were collected from the instrumented treadmill at 1,000 Hertz (Hz), low-pass Butterworth filtered at 35 Hz, and normalized to body weight. A 50 Newton (N) (Tirosh and Sparrow, 2003) threshold was used to determine initial contact and toe off for each step for the first 5 gait cycles (5 right steps and 5 left steps). Therefore, stance and swing portions of the gait cycle were determined when the vertical ground reaction force surpassed or fell under the 50 N threshold, respectively (Heiderscheit et al., 2011). Spatiotemporal running variables of step rate (steps per minute), step length (meters), and contact time (seconds) were calculated using a custom Matlab script (Mathworks Inc., Natick, MA, USA). Contact time was determined from the stance portion of the gait cycle when the foot is in contact with the ground. Step rate was calculated using the total time to complete 5 gait cycle to yield the number of steps per second, and then multiplied by 60 s to quantify the number of steps/minute (Goss and Gross, 2013). Step length was calculated by identifying the time difference (seconds) between initial contact of the left and right foot and multiplying that time by the treadmill speed (meters/second) to yield a step length distance in meters. OptoGait collected data at 1,000 Hz, and all spatiotemporal running variables were automatically calculated using OptoGait Version 1.11.1.0 software (Microgate, Bolzano, Italy). Data collection start times were synchronized to concurrently collect data from the OptoGait and instrumented treadmill from three 10 s trials during the final minute of running at a constant self-selected pace. Five consecutive right steps from the force plate data were analyzed in Matlab and compared to the right foot averages automatically calculated by the OptoGait software.

### Statistical Analyses

Data were input into Microsoft Excel, and the right foot data from the three trials were averaged per variable and carried forward for statistical analyses. Statistical analyses were completed using R version 3.4.4 with alpha set at 0.05. The level of agreement between the devices was analyzed using two-way random effects intraclass correlation coefficients with averaged measures [ICC (2,3)] for step rate, step length, and contact time. ICCs were interpreted as excellent (>0.90), good (0.75–0.90), or poor to moderate (<0.75) (Portney and Watkins, 2009). Absolute agreement was expressed by coefficient of variation of method error (CV_ME_) (Portney and Watkins, 2009) and 95% limits of agreement (LOA) using a Bland-Altman plot (Bland and Altman, 1986; Giavarina, 2015).

## Results

Means, standard deviations, and level of agreement are described in Table 1. Compared to the instrumented treadmill, OptoGait demonstrated excellent agreement for step rate [ICC = 0.99 (95% CI = 0.97–0.99)] and step length [ICC = 0.99 (95% CI = 0.99–0.99)]. Longer contact times were calculated by the OptoGait; however, good agreement [ICC = 0.83 (95% CI = 0.65–0.92)] between the OptoGait and instrumented treadmill was demonstrated.

**Table 1:** Means, standard deviations, and limits of agreement of running parameters using OptoGait and instrumented treadmill.

	**OptoGait**	**Treadmill**	**ICC (2,3) (95% CI)**	**CV_**ME**_ (%)**	**95% LOA**
Step rate (steps/min)	172.44 ± 8.78	172.01 ± 8.80	0.99 (0.97–0.99)	0.85	−3.62–4.49
Step length (m)	1.126 ± 0.109	1.128 ± 0.115	0.995 (0.990–0.998)	0.97	−0.03–0.03
Contact time (s)	0.27 ± 0.03	0.24 ± 0.02	0.83 (0.65–0.92)	5.66	−0.01–0.07

*ICC, Intraclass Correlation Coefficient; CV_ME_, Coefficient of variation of method error; LOA, Limits of Agreement*.

**Figure 1:**
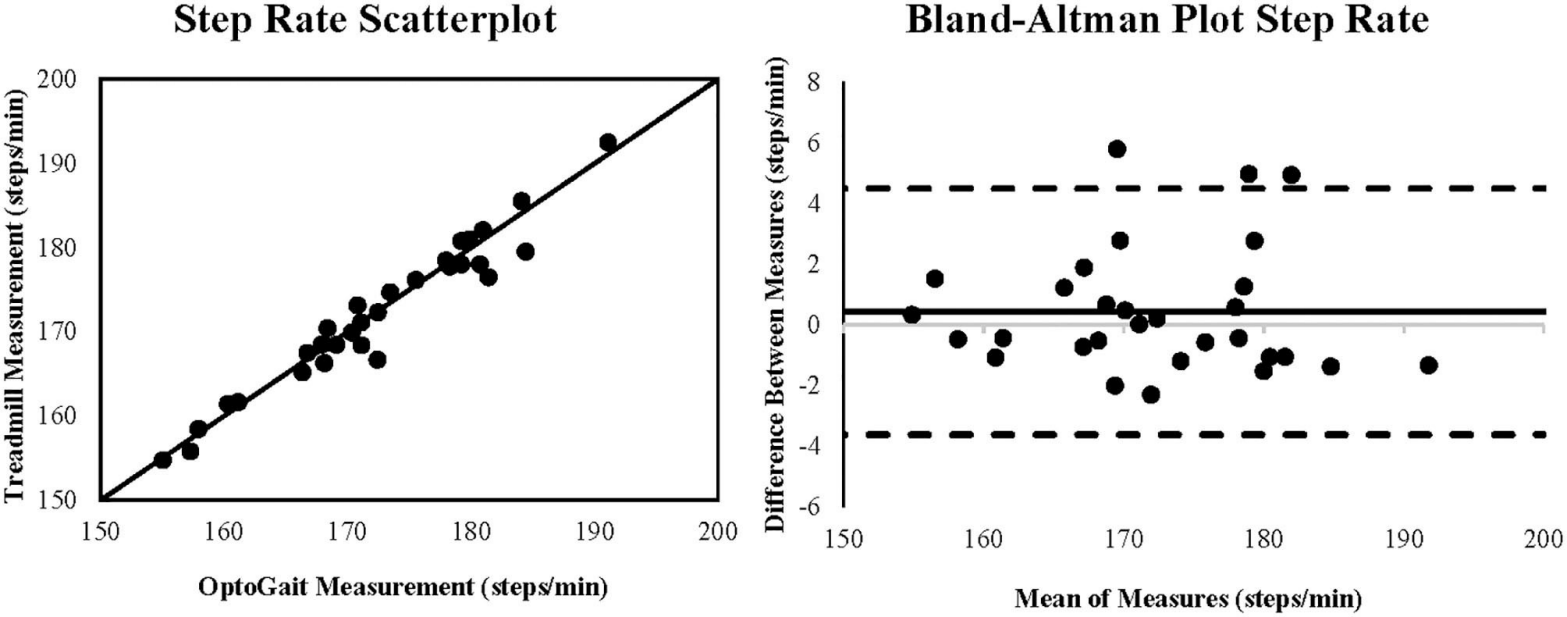
Scatterplot and Bland-Altman plot for the measurement of step rate (steps/minute) during running for the OptoGait vs. instrumented treadmill. The Bland-Altman plot includes the bias (solid black line) and the upper and lower 95% limits of agreement (dashed lines).

**Figure 2:**
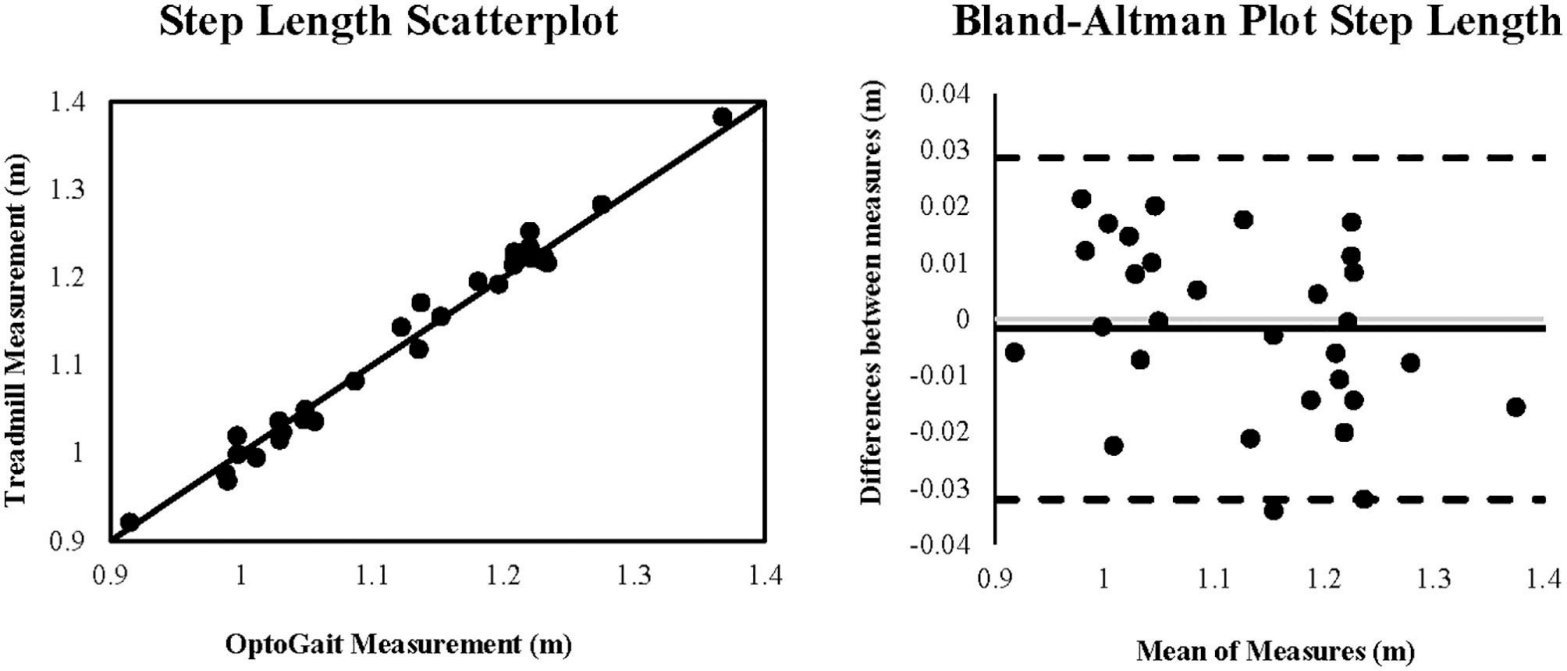
Scatterplot and Bland-Altman plot for the measurement of step length (meters) during running for the OptoGait vs. instrumented treadmill. The Bland-Altman plot includes the bias (solid black line) and the upper and lower 95% limits of agreement (dashed lines).

**Figure 3:**
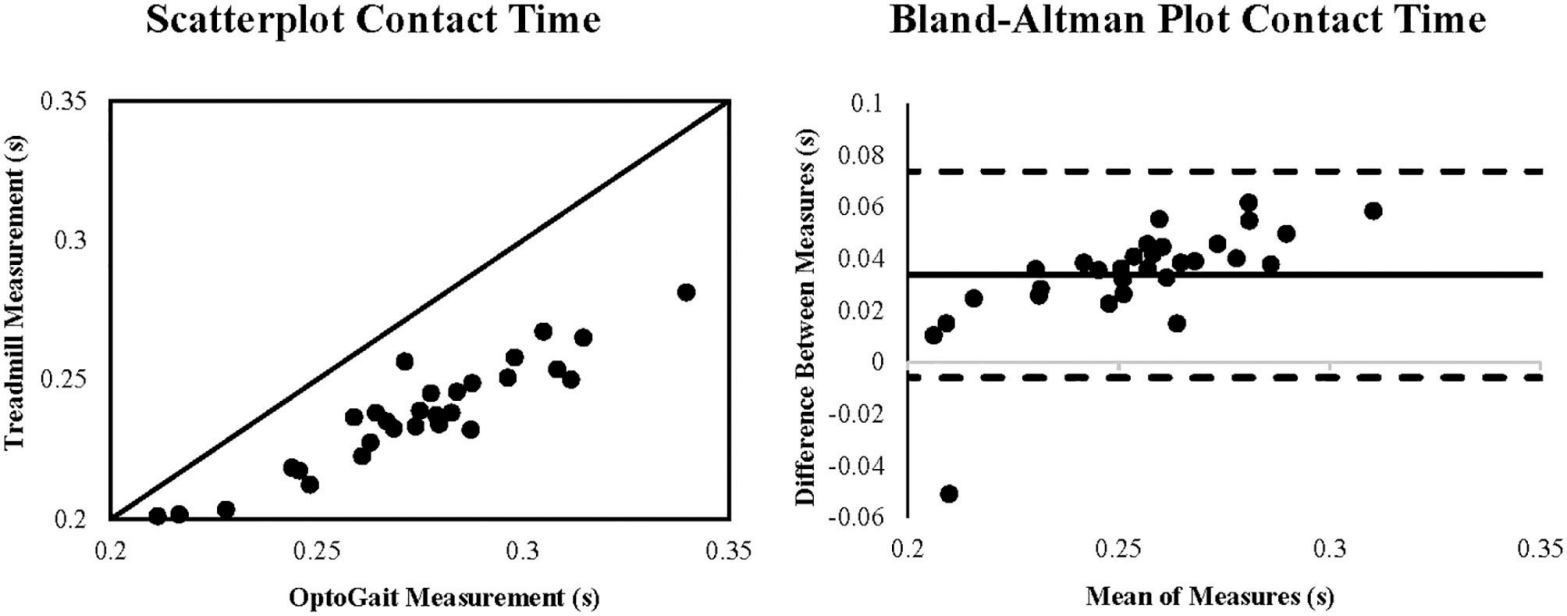
Scatterplot and Bland-Altman plot for the measurement of contact time (seconds) during running for the OptoGait vs. instrumented treadmill. The Bland-Altman plot includes the bias (solid black line) and the upper and lower 95% limits of agreement (dashed lines).

Coefficient of variation of method error (CV_ME_) was relatively small, ranging from (0.85 to 5.66%) with contact time demonstrating the greatest CV_ME_ as shown in Table 1. Scatterplots with lines of equality and Bland-Altman plots describing 95% limits of agreement are shown in Figure 1 for step rate, Figure 2 for step length, and Figure 3 for contact time. The scatterplot for contact time shows a right-sided skewness with OptoGait overestimating contact time. Bland-Altman plots and 95% LOA for step rate and step length are distributed in a symmetrical pattern; however, LOA for contact time are distributed in a positive pattern.

## Discussion

The purpose of this study was to investigate the level of agreement of the portable 1-meter OptoGait photoelectric system compared to an instrumented treadmill during treadmill running. The OptoGait demonstrates good to excellent agreement for measuring step rate, step length, and contact time during treadmill running for healthy runners.

Excellent agreement between the OptoGait and the instrumented treadmill was observed for step rate and step length during treadmill running. Previous studies have reported excellent OptoGait agreement for step rate and step length during walking (ICC = 0.93–0.99) (Lienhard et al., 2013; Lee M. et al., 2014; Lee M. M. et al., 2014; Gomez Bernal et al., 2016), which was consistent with the results for running gait. Clinical intervention focused on increasing step rate or decreasing step length by 5–10% may reduce biomechanical risk of injury (Edwards et al., 2009; Schubert et al., 2014; Adams et al., 2018). Additionally, interventions with primary or secondary aims of increasing step rate and/or decreasing step length have been used in patient populations with a history of patellofemoral pain syndrome (Davis et al., 2020), anterior cruciate ligament reconstruction (Bowersock et al., 2017), iliotibial band syndrome (Allen, 2014), and chronic exertional compartment syndrome (Helmhout et al., 2015) with observed levels of decreased joint loading, decreased pain levels, and improvements in self-reported outcomes. With the observed high level of accuracy of the OptoGait in calculating step rate and step length, clinicians could implement the OptoGait to objectively monitor changes in these gait parameters in response to gait retraining interventions.

Contact time demonstrated good agreement between the OptoGait and treadmill; however, the scatterplot reveals that contact time was overestimated for 29 out of 30 subjects on the OptoGait (Figure 3). This systematic bias may be attributed to the OptoGait photoelectric cells being 3 mm above the treadmill belt, suggesting OptoGait could detect a foot strike from disruption of light signals prior to the foot contacting the treadmill. The influence of this offset may be exacerbated by the 50 N force threshold used to determine initial contact and toe off on the instrumented treadmill. Although threshold ranges between 10 and 50 N can be used to define initial contact, lower thresholds may be more acceptable for lower walking speeds on embedded force plates (Tirosh and Sparrow, 2003). To reduce false foot strikes from the inherent noise of the moving treadmill belt (Zeni et al., 2008) at running speeds, a higher threshold for defining initial contact was used. Contact time on the OptoGait has demonstrated good to excellent agreement (ICC's = 0.85–0.99) for treadmill and over ground walking (Lienhard et al., 2013; Lee M. et al., 2014; Lee M. M. et al., 2014; Alvarez et al., 2017) with longer contact times reported by the OptoGait being attributed to the 3 mm offset (Lienhard et al., 2013). However, initial pressure threshold for foot strike was not defined by the authors in previous studies for the reference devices (Lienhard et al., 2013; Lee M. et al., 2014; Lee M. M. et al., 2014). For the present study, OptoGait recorded a 14.3% longer contact time, which is likely attributed to the 3 mm offset and the threshold for initial contact on the instrumented treadmill. Therefore, caution is warranted when comparing contact times between the two devices with OptoGait reporting longer contact times.

### Limitations

Although this study did not address OptoGait reliability, others have demonstrated good to excellent intra-rater and inter-rater reliability for spatiotemporal parameters of running gait (Jaén-Carrillo et al., 2020). Limitations of our study include self-selection of shoes and device filtering settings. The diodes may detect thick heel soles prior to exceeding the force threshold needed for initial contact on an instrumented treadmill. Individuals in traditional shoes (18 participants), defined as a heel to toe drop >8 mm, demonstrated an overestimated contact time by 15.7%, and individuals in minimalist or partial-minimalist shoes (12 participants) by 12.0%. These changes may have been more pronounced if further classification of partial minimalist and minimalist was defined.

Filter setting options in OptoGait are available to increase the minimum number of interrupted LED diodes for the start contact time (GaitR IN) and the completion of contact time (GaitR OUT). The predefined treadmill test utilized had a default filter setting of 0 LED. Although some studies have suggested using a filter option for optimizing spatiotemporal outcomes, the recommended number of LED filters is still debatable (Garcia-Pinillos et al., 2019; Healy et al., 2019). Further studies are needed to validate the need for filter settings for running gait.

## Conclusion

The present study demonstrates the agreement of the OptoGait as a method for analyzing spatiotemporal parameters of running gait. Manipulation of spatiotemporal parameters during running gait is a technique utilized to reduce biomechanical injury risk (Edwards et al., 2009; Heiderscheit et al., 2011; Schubert et al., 2014; Wellenkotter et al., 2014; Adams et al., 2018). The OptoGait may be utilized to objectively monitor changes in response to training program or gait retraining interventions. The OptoGait demonstrated good to excellent agreement in the evaluation of running step rate, step length, and contact time and should be considered for use in clinical, training, and research settings.

## Data Availability Statement

The raw data supporting the conclusions of this article will be made available by the authors, without undue reservation.

## Ethics Statement

The studies involving human participants were reviewed and approved by Keller Army Community Hospital Institutional Review Board. The patients/participants provided their written informed consent to participate in this study.

## Author Contributions

AW, EM, and DG designed the research protocol. AW, EM, and MJ performed data collection. GF wrote code for data analysis. AW, EM, GF, and DG analyzed and interpreted the data. All authors contributed to the final version of the manuscript.

## Conflict of Interest

The authors declare that the research was conducted in the absence of any commercial or financial relationships that could be construed as a potential conflict of interest.
